# The contact structure of Great Britain’s salmon and trout aquaculture industry

**DOI:** 10.1016/j.epidem.2019.05.001

**Published:** 2019-09

**Authors:** A.E. Jones, L.A. Munro, D.M. Green, K.L. Morgan, A.G. Murray, R. Norman, D. Ryder, N.K.G. Salama, N.G.H. Taylor, M.A. Thrush, I.S. Wallace, K.J. Sharkey

**Affiliations:** a University of Liverpool, UK; b Marine Scotland Science, Aberdeen, UK; c University of Stirling, UK; d Centre for Environment, Fisheries and Aquaculture Science (Cefas), Weymouth, UK

**Keywords:** Aquaculture, Network, Graph, Giant component, Fish diseases, Control policy

## Abstract

We analyse the network structure of the British salmonid aquaculture industry from the perspective of infectious disease control. We combine for the first time live fish transport (or movement) data covering England and Wales with data covering Scotland and include network layers representing potential transmission by rivers, sea water and local transmission via human or animal vectors in the immediate vicinity of each farm or fishery site. We find that 7.2% of all live fish transports cross the England-Scotland border and network analysis shows that 87% of English and Welsh nodes and 72% of Scottish nodes are reachable from cross-border connections via live fish transports alone. Consequently, from a disease-control perspective, the contact structures of England and Wales and of Scotland should not be considered in isolation. We also show that large epidemics require the live fish movement network and so control strategies targeting movements can be very effective. While there is relatively low risk of widespread epidemics on the live fish transport network alone, the potential risk is substantially amplified by the combined interaction of multiple network layers.

## Highlights

• 87% of English and Welsh nodes are reachable from Scotland via the live fish movement network.
• 72% of Scottish nodes are reachable from England or Wales via the live fish movement network.
• 7.2% of all live fish movements cross the England-Scotland border.
• Targeted surveillance on a handful of sites is effective in identifying and controlling outbreaks.
• The combination of different mechanisms of transmission increases the chance of large epidemics.

## 1 Introduction

Finfish aquaculture in the United Kingdom continues to increase, with the UK-wide collated figures for 2014 reported to be 193 kt (Hambrey and Evans, 2016) with an imputed value of £762 m. The dominant production species by tonnage and value is Scottish marine farmed Atlantic salmon (*Salmo salar*) which accounted for 163 kt in 2016 (Munro and Wallace, 2019). In 2017, salmon was the UK’s most valuable food export (Federation, 2018) with the industry providing employment in remote and rural communities. A limiting factor in the sustainable expansion of the aquaculture industry is the management of infectious diseases which can lead to diminished production. For example, in a study of one marine Atlantic salmon farming company’s production data, an estimated one third of salmon mortality was attributed to infectious disease (Kilburn et al., 2012).

Finfish diseases can have severe economic and social impacts on aquaculture. From 2007 to 2009, an outbreak of infectious salmon anaemia virus (ISAV) in Chile had a direct economic impact estimated at US$2bn, with a loss of 15,000 jobs (Mardones et al., 2011). Thus, understanding potential routes of disease transmission and developing effective control measures is paramount in ensuring a sustainable industry. Eight finfish diseases are notifiable in the UK under European Council and Aquatic Animal Health Regulations (Council of the European Union, 2006; Anonymous, 2009a, b). Of these, five have previously occurred in the UK: viral haemorrhagic septicaemia (VHS), infectious salmon anaemia (ISA), bacterial kidney disease (BKD), koi herpesvirus disease (KHV), spring viraemia of carp (SVC) (Cefas, 2014). Controls, including the restriction of live animal movements, may be applied to premises and river catchments where notifiable disease is suspected or confirmed to be present (Anonymous, 2009a, b).

The use of network analysis in understanding livestock industries and their potential for sustaining epizootics is well-established (Dent et al., 2011; Robinson et al., 2007; Christley et al., 2005). In aquaculture, this has also been investigated (Green et al., 2009, 2012; Ruane et al., 2009; Munro and Gregory, 2009; Jonkers et al., 2010; Yatabe et al., 2015) and spread of diseases such as ISAV and BKD have been associated with specific movements of fish and other contacts between sites (Murray et al., 2002, 2012; Mardones et al., 2014). A feature of the British aquaculture industry is that, for the control of notifiable diseases, it is divided administratively. Statutory responsibilities are delivered by Fish Health Inspectorates owned respectively by the Centre for Environment, Fisheries and Aquaculture Science (Cefas) in England and Wales; and by Marine Scotland Science (MSS) in Scotland. Despite the connectivity of these two administrative regions via the transport of live fish, to date there has been no comprehensive study of the combined network. Here we describe the first unified framework of Great Britain’s (GB) salmonid aquaculture industry to provide a foundation for basic network analysis and epidemiological outbreak simulations.

Network theory has applications across science and engineering and in recent years there has been considerable interest in the analysis of “multi-layer” networks; these are networks which encompass different types of connections and different types of nodes and incorporating such subtleties is important for improving our understanding of complex systems (Kivelä et al., 2014). Disease transmission within the salmonid industry is a prime example of such a system: nodes of the network are farms or recreational fishing waters which hold trout or salmon. Fish farms are authorised to rear fish for ongrowing, restocking fisheries or for direct sale to table market. Recreational fishing (angling) waters (fisheries) range from small put and take lakes to open stretches of river. Links in the network through which diseases can be transmitted occur in several layers due to link types spanning the socio-economic, the ecological and the environmental. Previous studies have primarily focused on potential transmission via networks of live fish movements (e.g.(Green et al., 2012; Yatabe et al., 2015)). A multi-layer network of the salmonid industry of England and Wales, incorporating transport, river and local links (transmission via human or animal vectors in the immediate vicinity of each site) was the basis for the epidemiological modelling study of (Jonkers et al. (2010)). Here, we extend this multi-layer network approach to include Scotland. In our combined network, we consider four layers with different link types: movement of live fish between sites, waterborne pathogen transmission via the river system, waterborne transmission in the marine environment, and local transmission via human or animal vectors in the immediate vicinity of each site.

## 2 Data and methods

### 2.1 Geographical area and time period

Live fish movement data was provided by Cefas and MSS for England and Wales (2011–2013) and Scotland (2009–2011) respectively. Geographically, our analysis is confined to GB (England, Scotland, and Wales, including their component islands), excluding the Isle of Man, the Channel Islands, and Northern Ireland. GB and the island of Ireland are considered as separate epidemiological units (Yatabe et al., 2015; Wiens, 2011) and therefore Northern Island is excluded from the network analysis. This separation is supported by considering pathogen distribution; salmonid BKD is widespread in GB but absent from Ireland (Wiens, 2011), and the same was true for infectious pancreatic necrosis virus until recently (Ruane et al., 2009). Furthermore, GB’s aquaculture industries are free of several notifiable pathogens which are widespread in much of continental Europe including infectious hematopoietic necrosis virus (IHNV), viral hemorrhagic septicemia virus (VHSV), ISAV (but not HPR0 variant) and the monogenean parasite *Gyrodactylus salaris* (Manual of diagnostic tests for aquatic animals, 2016; Dalgaard et al., 2004). GB is therefore a reasonably self-contained epidemiological unit suitable for analysis.

### 2.2 Fish species groups

We focus on two salmonid species groups, referred to as salmon and trout, where salmon include Atlantic salmon (*Salmo salar*) only, and trout refers to all other salmonid species stocked (genera: *Salmo, Oncorhynchus, Salvelinus, Coregonus, Thymallus*), here almost exclusively rainbow trout and brown trout.

### 2.3 Sites

We define our network nodes as single-group; if both salmon and trout nodes are in close proximity and are jointly managed, they form a multi-group site. We considered both fish farms and recreational fisheries. For the purposes of this analysis, sites in England and Wales were categorised as fish farms if they are authorised for operation by Cefas and subject to surveillance. In Scotland, fish farm sites were categorised by consultation with MSS. To ensure only active sites were included in the network, non-farm sites (categorised as “other”) were only included in the analysis if they were a source or target of a live fish movement. Sites that registered only live fish movements peripheral to the industry, such as restocking-only facilities, were excluded.

### 2.4 Live fish transport network

Transport of fish by road, shipping and air includes movement of fingerlings from hatcheries to on-growing sites and fishery stocking, together with the occasional movement of fish to processing facilities. These processes can potentially transfer pathogens carried by fish, water and/or equipment (e.g. tanks, nets) (Jonkers et al., 2010; Murray et al., 2002). Due to the risk of disease spread, EU member states are required to record live fish movements under EU directive 2006/88/EC (Council of the European Union, 2006; Green et al., 2009, 2012; Munro and Gregory, 2009). Fish farmers therefore have a legal obligation to keep records of all movements of live fish on and off their premises, and to make this information available to the competent authority (i.e. Cefas or MSS Fish Health Inspectorates in GB).

Prior to 2014 (thus including the data we consider), movements of live fish to recreational fisheries in England and Wales required consent from the Environment Agency under Section 30 of the Salmon and Freshwater Fisheries Act (Anonymous, 1975). Section 30 farm-to-fishery transports required individual approval specifying species being moved and a dated time window (up to six weeks) during which one or more movements were to be carried out. By contrast, farm-to-farm transports are dated only by year, and records do not include species. As fish transports are by definition group-specific in our network reconstruction, this information was inferred based on the species group being stocked by the farms concerned.

In Scotland, under the Aquatic Animal Health (Scotland) Regulations 2009 (Anonymous, 2009a), aquaculture production businesses are required to keep records of live fish movements onto and off their fish farms (Werkman et al., 2011, 2014). These official records are held by the Fish Health Inspectorate at MSS, and are dated and recorded at both source and destination farms, providing overlap and confirmation opportunities. Scottish paper records covering 2009–2011 were transferred to an electronic database. This was done according to the validation process used in previous network analysis of fish movement data in Scotland (Green et al., 2009, 2012; Munro and Gregory, 2009), with only confirmed movements (i.e. only those movements recorded at both source and destination sites) being entered into the database. The 2009–2011 dataset was the most complete data available at the time. Live fish movements that cross the English-Scottish border were also included in this study. Both agencies recorded movements in both directions across the border. Here, Scottish data was the primary source of information on cross-border connections, since MSS records include the specific Cefas site involved. Cross-border links were cross-checked with Cefas records where possible and also cover the period 2009–2011.

Our objective was to provide a robust representation of the links within the network. For salmon, a significant number of sites take two years to get from input to harvest and fallow periods can last for up to a year (Munro and Wallace, 2019). The production cycles for trout are typically less than this. Consequently a three year timeframe captures the full industry structure while minimising the potential for changes in that structure due to an over-long time period. While it would be ideal to have overlapping years, the offset between Cefas data and MSS data, necessitated by data availability, does not interfere with the objective of characterising the industry structure.

### 2.5 River links

Some fish diseases can be maintained in wild populations with examples in parasitic disease or sustained via vertical transmission. However, most evidence points to the threat to wild fish in the vicinity of fish farms and not the threat posed to fish farms from wild fish (see Jonkers et al. (2010)). Our primary concern is notifiable infectious diseases with the potential for epidemic spread rather than endemic infectious diseases. Environmental reservoirs sustaining endemic infection are therefore explicitly excluded here, although this background risk could be incorporated in infectious disease simulation models and this would be relevant for the parasite *Gyrodactylus salaris* once it is endemic.

Between-site river connections were derived from the European Environment Agency’s *European Catchments and River Network System* (ECRINS (Eea, 2012)), from which we extracted 20,578 river segments that comprise the river systems in mainland Britain (Jonkers and Sharkey, 2016). This provides the basis for constructing downstream waterborne transmission links between sites that discharge into, or take in water from, any river or stream in Britain (as opposed to the use of boreholes, mains water, sewage discharge, and recirculation systems). We note that for Scottish fisheries, only intake was recorded; here discharge was assumed to match intake, which, from a transmission risk perspective, represents the worst-case scenario.

Each site with inflow or outflow was associated with the nearest point on the nearest ECRINS segment, with a maximum distance to river of 2 km enforced to account for the spatial resolution of the ECRINS data and ensure the correct river segment was associated with each site. Subsequently, sites that discharged waste water into the river were considered starting points for following that particular river branch through all confluence junction points down to the sea, cataloguing all other river-connected sites along the way, provided they take in water from the river. Each of these constituted a destination site for a river link, given the start point’s site as source, for which downstream distance between source and destination was also stored.

Cross-border links are possible via two rivers, the (Cumbria) Esk and the Tweed, which straddle the England-Scotland border. However, aquaculture in the Esk catchment falls entirely under the jurisdiction of Cefas, whereas MSS is responsible for the entire Tweed catchment, and hence our analysis of cross-border links consider only those within the transport network layer.

### 2.6 Marine links

Transmission of disease may also occur at sea via the dispersal of pathogens by water currents. A network was constructed by applying a modified hydrodynamic expression for the movement of pathogenic agents in the marine environment (Salama and Murray, 2011, 2013). This model was parameterised using observational data of current speed and direction collected during the site licensing process by the Scottish Environment Protection (Agency (2008)). The modelling of this hydrodynamic contact network is described in the appendix. No similar network exists for England or Wales, since open net pen marine farming of finfish does not occur in these countries.

### 2.7 Local links

We defined bi-directional local links, representing fomite transmission via animal vectors such as eels and piscivorous birds, as existing between all sites within a 3 km radius. This distance threshold was based on an existing epidemiological simulator for aquaculture disease (Jonkers et al., 2010), which uses a two-dimensional Gaussian diffusion kernel for which transmission likelihood drops steeply with radial distance from the source, reaching half of its maximum at 833 m and is effectively zero at 3 km.

### 2.8 Network analysis metrics

We first describe the structure of the network according to the characteristics of sites and/or nodes (location, species group, site type, freshwater or marine) and links (link type, cross border or within-authority, link distance). We then consider the number of nodes at risk of infection via different network layers by calculating sizes of network subcomponents (Newman, 2018).

The out-component of a node is the set of other nodes which can be reached by following all network links from the node, including the node itself. Similarly, the in-component is the set of nodes from which the node can be reached. The “largest strongly-connected component”, LSCC, is the largest group of nodes in the network which are strongly-connected (every node can be reached from every other node). This gives an indication of the potential for endemic or self-sustaining epidemics and is also a measure of the probability of large epidemics, since large epidemics would start in the LSCC. We also consider the size of the network consisting of all nodes outwardly reachable by from the nodes in the LSCC (denoted LSCC + out), which gives the maximum outbreak size for introduction into the LSCC.

To quantify outbreak risk due to network structure, we define an additional metric, the “expected outward reach” or EOR. For a given node, the term “reach” (Green et al., 2009), is defined to be the number of nodes that can be reached by following network paths emanating from this node and including the node itself (the size of the out component). For nodes in the LSCC, the reach is equal to the size of LSCC + out. The EOR is then the expected value of this quantity for nodes selected uniformly at random. This is the expected maximum number nodes infected, given a uniformly seeded infection event and so gives a metric for the worst case epidemic scenario given the network structure.

In addition to exploring this measure of network connectivity for different network layers, we consider the impact of control measures, in the form of a complete ban on live fish movement for selected nodes, on the EOR of the resulting network. This gives an indication of the extent to which the network can be broken up, and consequently disease spread reduced, by removing nodes which are influential within the network. In graph theory, this influence, or “centrality” has no single definition, but rather a number of alternative measures, the usefulness of which depends on the specific application. Here we consider three different centrality measures for selection of significant nodes:

1 The number of incoming links to a node, or inward degree.
2 The number of outgoing links from a node, or outward degree.
3 The betweenness of a node, defined as the number of shortest paths between pairs of nodes which pass through a given node (Newman, 2018), thereby having the potential to identify nodes which are important links between sub-components of the network.

The first two are locally determined measures and so are advantageous because they can be determined without full knowledge of the network structure. Betweenness is found to be the most effective out of many measures investigated. The impact of removing the nodes according to each criterion is calculated by sequentially removing the node from the transport network layer that has the greatest centrality and then recalculating the full network EOR. The centrality measures for the reduced network are then determined to identify the relevant node for the next iteration. Fifty nodes are removed in this way.

To quantify the spatial pattern of transmission risk on the network, we also map the mean size of the in-component and out-component of the nodes located within cells of a 10 km x 10 km grid over GB, and contrast the component sizes for the transport network layer and the full network.

## 3 Results

### 3.1 Sites, species group and nodes

**Fig. 1:**
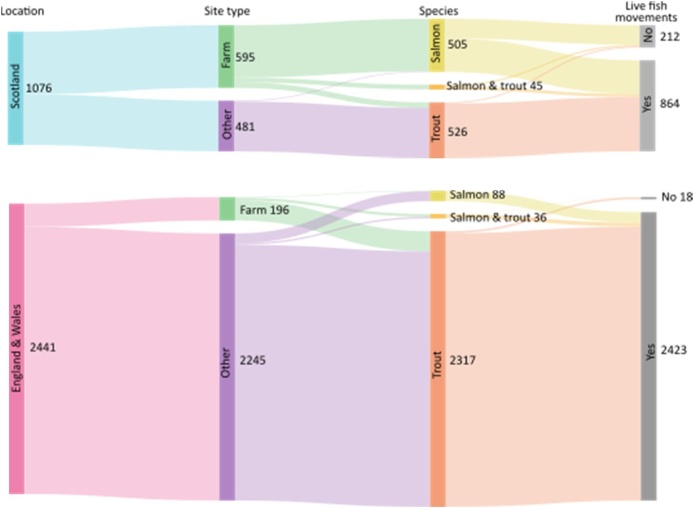
Breakdown of the 3517 geographical sites by location, site type, species group and presence of live fish movement records in the database. Site count breakdown for species group (multi, salmon, trout) is as follows:. England and Wales: 2441 total with 196 farms (23,5,168), 2245 other (13,83,2149); Scotland: 1076 total with 595 farms (44, 498, 53), 481 other (1, 7, 473).

The network representation of the salmon and trout aquaculture industry in GB comprises 3517 geographical sites, of which 791 are interpreted here as farm sites. The England and Wales sub-network contains 2441 distinct sites, and the Scottish sub-network comprises 1076 distinct sites. A subset of 3287 sites have transport records in the database: 2423 in England and Wales and 864 in Scotland. These can be further broken down into designated farm sites and other sites as illustrated in Fig. 1.

A distinct feature of the Scottish network is the presence of a large number of marine farm sites that mostly (94%) produce salmon (Table S2b). Over 34% (369) of Scottish sites are marine, associated for control purposes with a marine management area rather than a river catchment. These marine sites are large: 75 of the 139 salmon sites that produced salmon in 2015 harvested over 1 kt (Munro and Wallace, 2019). Marine sites are present in both the Scottish salmon and trout industries.

### 3.2 Structure of the live fish movement network

For the purposes of describing the network structure, we consider network nodes rather than geographical sites, such that each of the 81 multi-group sites (those with records of both salmon and trout) are treated as two separate nodes in close proximity. The live fish movements within Scotland comprise 1669 unique links between nodes, carrying a total of 15,471 transports over three years. In Scotland, 984 of the links are between farms. In England and Wales, there are 3293 unique links, carrying a total of 9898 transports with 583 of the links being between farms.

Table S3c in the appendix shows an alternative breakdown of Scottish links by water type and destination site type, distinguishing between inland transports, marine transports, and those that cross the coast in either direction; these latter movements heavily favour the outward route, from land to sea, corresponding to supply of smolts. Almost all movements involving marine sites are to farms only.

**Fig. 2:**
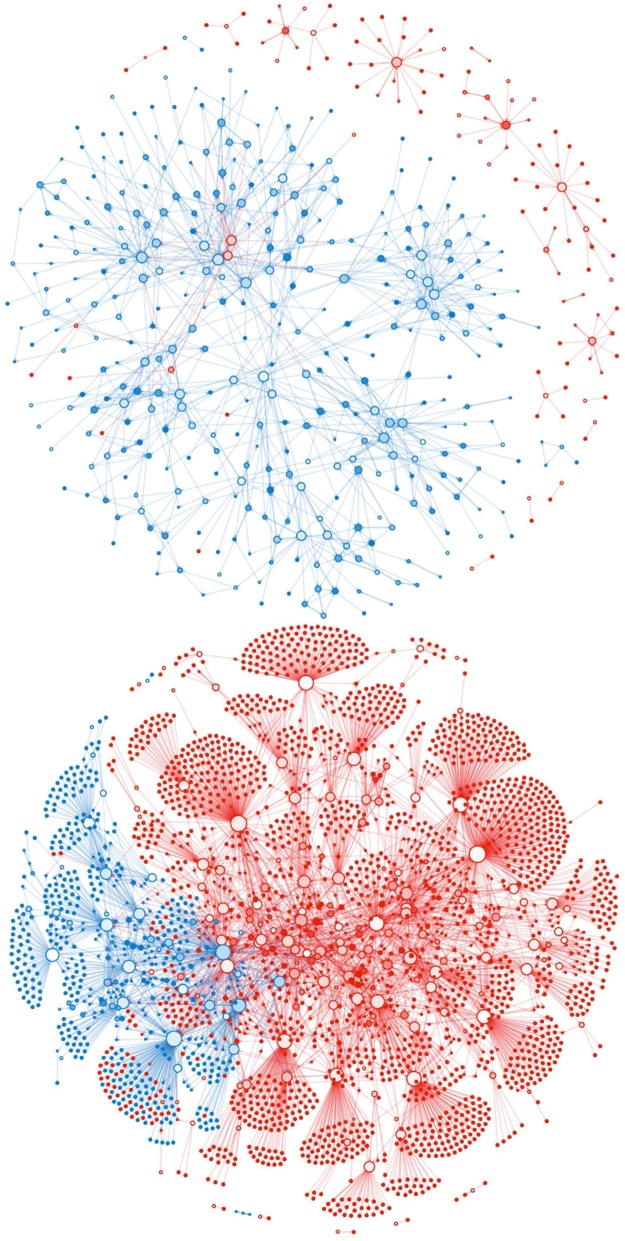
Plot of the British salmonid aquaculture industry live fish movement networks for salmon (top), and trout (bottom) species groups. Nodes in the Marine Scotland network are blue and nodes in the Cefas network red. Node size is proportional to the log of the number of connections. Node color scales to white depending on proportion of connections for which node is the source (i.e. white = source, full color = sink). Links have the colour of the source node. (For interpretation of the references to colour in this figure legend, the reader is referred to the web version of this article).

Fig. 2 illustrates the structure of GB’s salmonid aquaculture industry in terms of live fish movements, with nodes colour-coded by location, and including only those 3326 (of a total of 3598 nodes across both species groups) connected via live fish movements to the main clusters during the period considered. Since movements are species group-specific, the network is depicted as two separate clusters, one for each group. For both groups there are smaller clusters of nodes that are not connected via live fish movements to the main clusters. The hub-spoke nature of the trout industry is conspicuous in Fig. 2, where the trout hub sites are primarily farms (see Fig. S1). In general, this hub-spoke structure is not seen in the salmon industry due to the lack of salmon fisheries, although there are a few exceptions, corresponding to stock enhancements of salmon in England and Wales.

### 3.3 Cross-border transport network

Collated cross-border transports for the years 2009–2011 yielded number of links per species group and destination type, summarised in supplementary Table B3a. These involve 143 unique nodes in England and 59 in Scotland (total node numbers are in supplementary Tables E1 and S1a respectively). The total number of border-crossing connections (287) represents 5.5% of the network’s total transport infrastructure. When added to each agency’s accountable transport network, they represent 8.0% of Cefas-administered transport links, and 14.7% of MSS-administered transport links. Another notable feature is the dominance of trout links in both directions (243 links out of 287, or 85%). There are 150 cross-border links from England and Wales to Scotland, with 813 transports, and 137 cross-border links from Scotland to England and Wales, with 1164 transports.

Whereas the majority of movements within a single authority are within 200 km, cross-border movements have more longer-range transports (Fig. S3), particularly those from England and Wales to Scotland, including salmon transport from north western England to Shetland and eastern England to northern Scotland, and trout transport from southern and south-western England to southern Scotland (not shown due to data confidentiality). The cross-border network is depicted in Fig. S2.

Supplementary Table B3b details the 1977 cross-border live fish movements for the GB-wide transport network through the links listed in Table B3a. Cross-border transports represent 16.6% of movements when added to the English and Welsh internal ones (in the period 2011–2013), and 11.3% if added to the ones within Scotland over 2009–2011. As a proportion of the total salmonid aquaculture traffic in GB, cross-border transports make up 7.2% of all live fish movements.

Analysis of the onward network links reveals that the 115 nodes in England and Wales with cross border connections from Scotland can reach a further 2057 nodes in England and Wales via transport links. A total of 87% of the Cefas network nodes are therefore reachable from Scotland. In the opposite direction, the 50 Scottish nodes with connections from England and Wales can reach a further 630 nodes; or a total of 72% of the MSS network is reachable from England and Wales via transport links. Including all link type layers (see below) in the onward network increases these figures to 95% of Cefas nodes and 87% of MSS nodes.

### 3.4 Combined network

**Fig. 3:**
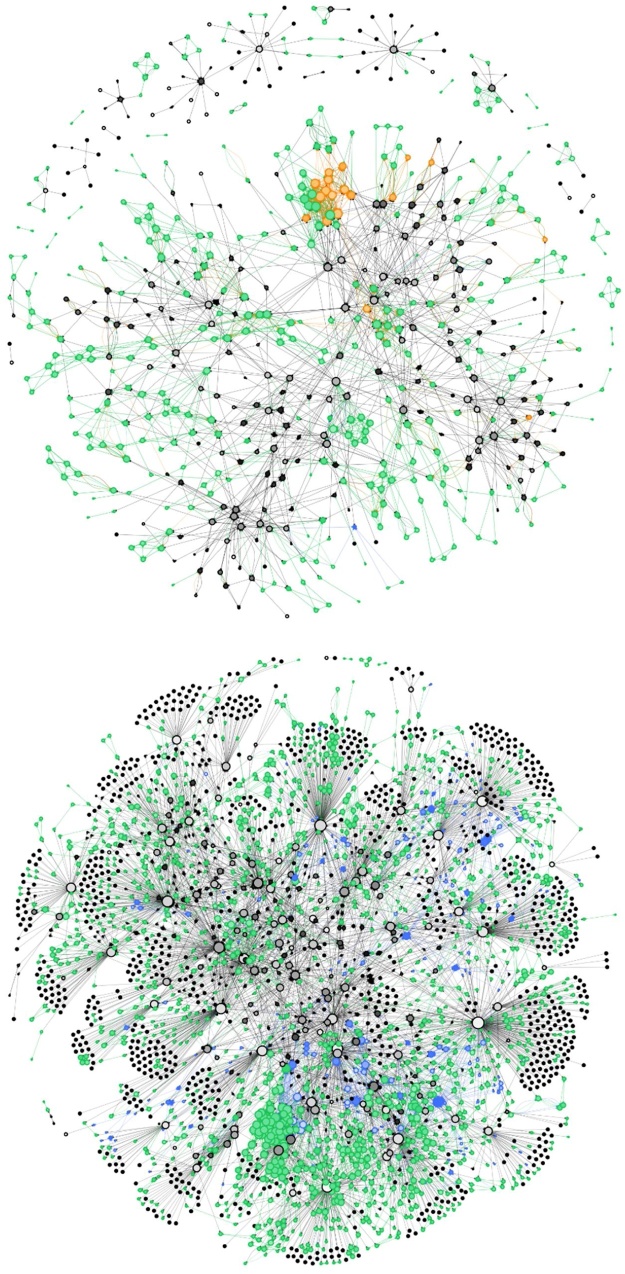
The multi-layer network of the GB salmonid aquaculture industry for salmon (top), and trout (bottom). Link layers are colored by type: blue = river, local = green, transport = black and marine = orange. Nodes are colored by their dominant link type. Node color scales to white depending on proportion of connections for which node is the source (i.e. white = source, full color = sink). Links have the colour of the source node. (For interpretation of the references to colour in this figure legend, the reader is referred to the web version of this article).

Combining all link type layers results in a highly complex network (Fig. 3). Supplementary Table B4 describes the distribution of link distances by type. Live fish movements span the longest distances by far (mean of 90.1 km). The next longest are river links (mean of 19.7 km), followed by marine links (mean of 3.9 km). Local links are constrained by the modelled cut-off at 3 km. The longest link in the network is a cross-border transport spanning 793 km. Links between the Cefas and the MSS networks are found only in the transport layer.

The diameter of the network is given by the longest shortest path between nodes. For the combined network, there are three paths with the longest path length of 22 links. These three paths differ only by one node each and have the same breakdown of links including three of the four network layers: 10 local links, 0 river links, 11 transport links and 1 marine link. Table 1 shows the combined network broken down by links and nodes which are present only in specific layers and combinations of layers. For the local, river and transport layers, the majority of links are present only in those layers; for example, 5625 out of 6250 (90%) of local links are unique to the local layer and 4925 out of 5249 (94%) of transport links are unique to the transport layer. In contrast, the marine layer contains only 143 links out of 382 (37%) which are unique to that layer.

**Table 1:** Number of unique links and unique nodes present only in single network layers and combinations of network layers, together with the total number of links and nodes within the specified layers which are present in the network.

Network layers	Unique links	Total links	Unique nodes	Total nodes
L + R + T	24	24	347	347
L + T + M	37	37	122	122
**Total (3 layers)**	**61**		**469**	
L + R	137	161	10	357
L + T	234	295	1514	1,983
L + M	193	230	8	130
R + T	20	44	156	503
R + M	0	0	0	0
T + M	9	46	9	131
**Total (2 layers)**	**593**		**1,697**	
L	5625	6250	201	2,202
R	658	839	4	517
T	4925	5249	1,178	3326
M	143	382	0	139
**Total (1 layer)**	**11,351**		**1383**	

The size of the largest strongly connected component (LSCC) for each subnetwork is given in Table 2. By definition, the group-specific transport network structure separates the movements in the salmon industry from those in the trout industry and so these should initially be considered separately. The movements in the salmon industry lead to an LSCC of 54 and those in the trout industry lead to an LSCC of 104. These are relatively small connected components. For the river network on its own, transmission is assumed to only go downstream; hence its LSCC has size 1. For local transmission (within 3 km) the LSCC is 79, and for marine waterborne links it is 17. Each of these layer LSCCs contribute to the LSCC of the combined network. Analysing the 725 nodes in the combined LSCC subnetwork further: for transport, other than the LSCC itself, the second largest component is 54 nodes (which is the salmon network LSCC), with all other components of size 8 nodes or less; for the marine links, all other components smaller than the LSCC are of size 4 nodes or less; for the local network, the other components are slightly larger on average, varying in size from 1 to 20 nodes.

**Table 2:** The size of the largest strongly-connected component (LSCC) as well as LSCC + out is given as the number of nodes out of a total of 3598 in the network (674 salmon and 2924 trout). Metrics are given for different combinations of link type layers, sorted in descending order by LSCC. Layers are transport (T), river (R), local (L) and marine (M). Additionally, %farm for LSCC and LSCC + out give the percentage of the nodes in these networks which are classified as farms. EOR is also given to the nearest whole number of nodes. Note all combinations other than transport are across both salmon and trout groups.

Network layers	LSCC	%farm (LSCC)	LSCC + out	%farm (LSCC + out)	EOR
All Layers	725	55	3419	21	740
T + R + L	722	55	3419	21	737
T + L + M	659	59	3416	21	675
T + L	656	59	3416	21	672
T + R + M	216	81	3,049	16	237
T + R	141	72	3,033	16	158
**T**	**104**	**91**	2572	**7**	**108**
T + M	104	91	2572	7	180
L	79	14	79	14	8
R + L	79	14	79	14	9
L + M	79	14	79	14	9
R + L + M	79	14	79	14	10
M	17	100	33	100	4
R + M	17	100	33	100	7

The LSCC increases significantly when we combine the network layers. Table 2 illustrates different layer permutations. In particular, it is clear that the combination of transport and local link layers yields a major change in the size of the LSCC, well beyond the combined individual LSCCs of the salmon and trout networks. Only relatively minor increments to the LSCC are provided by adding river and marine layers. It is the combined effect of all of these potential routes of transmission, but particularly of transport and local routes, that yields an epidemiological system that has a significant probability of propagating large outbreaks. Considering the transport network layer alone, 91% of nodes in the LSCC are farms (Table 2). This percentage decreases as other layers are added, reflecting the increasing importance of fisheries, reducing to 55% for all layers.

The fourth column in Table 2 (LSCC + out) includes all nodes reachable by the specified link types from the nodes in the LSCC. It shows that unlike the other transmission mechanisms, the transport network layer is by itself sufficient to spread a pathogen network-wide, potentially infecting 2572 trout nodes: 88% of trout nodes or 71% of all 3598 network nodes. Adding local transmission to the transport links (row 4 in the table) raises this worst-case proportion to 3416 nodes or 95% of all nodes. This is only marginally increased to 3419 nodes for all links types.

**Fig. 4:**
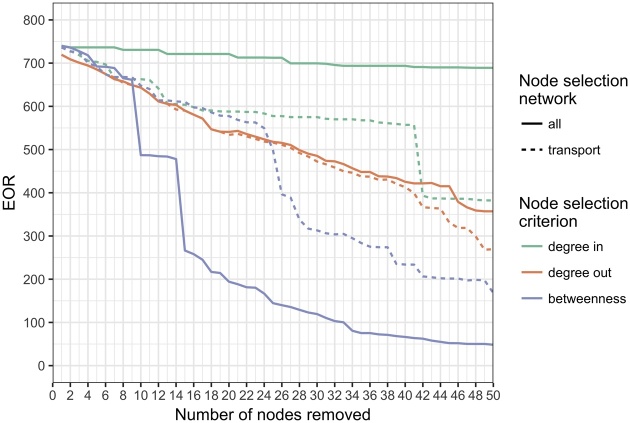
Impact on expected out-component size, EOR, of removing an increasing number of highly connected nodes from the transport layer. Nodes are selected at each step by ordering according to different criteria as defined in the Methods section: by degree in (green), degree out (red), or betweenness (blue), calculated on either the transport network layer (dashed line) or the full network (solid line). (For interpretation of the references to colour in this figure legend, the reader is referred to the web version of this article).

The EOR for transport links is 108 nodes or 3% of all nodes, whereas the EOR for all link type layers is 740 nodes or 21% of all nodes. This measure, reflecting maximum possible outbreak size, shows that the average size of an epidemic can be substantially increased by combining all network routes together. The impact of a small number of highly-connected nodes on the reach of the full network is illustrated in Fig. 4, which shows the resulting EOR when increasing numbers of highly-connected nodes are removed from the transport layer, with the nodes selected according to different estimates of their influence on the network. The most effective method for node selection is betweenness centrality calculated on all network layers, reflecting the presence of key nodes which connect sub-components of the network. Further analysis of the reduced networks depicted in Fig. 4 shows that by removing transport links from 15 nodes (0.4% of all nodes) using this criterion, the EOR for the combined network was cut to 36% of its value for the full network (EOR of 266 versus 740). By increasing this to 25 nodes (0.7%) using the same criterion, the EOR was reduced to 20% of its full value (EOR of 145). The 25 nodes selected in the latter case comprise four salmon nodes in Scotland, five trout nodes in Scotland, 1 salmon node in England and Wales and 16 trout nodes in England and Wales. Of the 25 nodes, 18 have cross-border links.

### 3.5 Spatial patterns of transmission risk

**Fig. 5:**
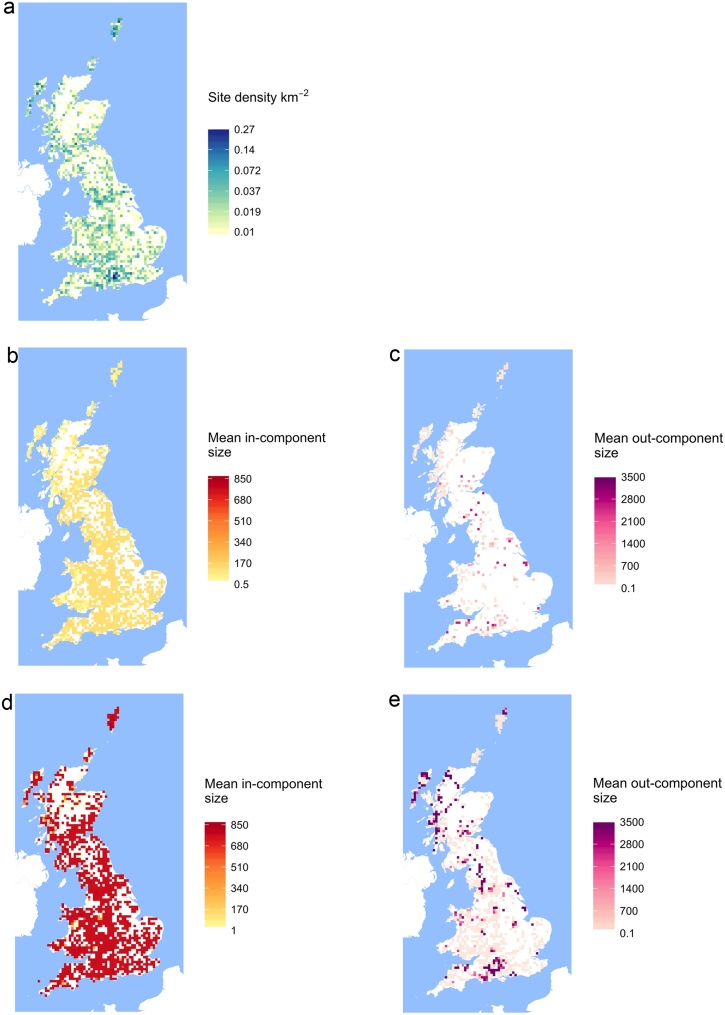
Maps of site density on a 10 km grid (a) and spatial patterns of mean in-component size (b, d) and out-component size or reach (c, e) for sites in each grid cell, for transport (b, c) and combined network (d, e).

The spatial density of sites in the network is illustrated on a raster grid of 10 km in Fig. 5, together with the mean size of the in and out components of nodes in these 10 km grid cells. The results for the network consisting of only transport links (middle row in Fig. 5) show that, for cells where sites are present, all locations are similarly reachable from other sites: there is a fairly homogenous distribution of in-component size across the country, with the majority of grid points having nodes reachable from around 130 other nodes. In contrast the out-component size is highly heterogeneous, with a large number of grid cells having a mean size of zero, and a scattering of cells with very large out-component size across south western England, northern England and south and central Scotland. When all links type layers are included in the network, the size of the in-component is increased by approximately a factor of 6 (median value = 764), remaining fairly spatially homogeneous. Out-component size also increases, with more grid cells having non-zero size. The number of cells with very large out-component sizes also increases, with 5% of grid cells having a mean size of more than 3418 nodes. In addition to an expansion of the large out-component area centered on Hampshire in the south of England, there is also a notable increase in the out-component size of grid cells in Scotland, particularly the west coast and the Outer Hebrides. Both are areas of high site density (Fig. 5a).

## 4 Discussion

We constructed the first island-wide representation of the structure of GB’s salmonid aquaculture industry, assimilating data covering England and Wales with data covering Scotland, representing a snapshot of the industry circa 2011. This is a comprehensive representation of the salmon and trout fish farms and fisheries of GB, overlaid with a detailed multi-layer network mapping of the potential routes of infectious disease transmission. These layers are live fish movement, transmission along rivers, transmission in the local area around sites, as well as diffusion and transport in the marine environment.

From a management perspective, responsibility for the control of serious diseases in aquatic animals in GB is divided into Scotland (MSS), and England & Wales (Cefas). Our results demonstrate the essential interconnectedness of these two administrative regions via the transport layer alone. A total of 7.2% of all live fish movement events go across the border, the vast majority of which are trout (93%). Furthermore, via the transport network alone, 87% of Cefas network nodes and 72% of MSS network nodes are reachable from cross-border connections. The importance of cross-border linkages demonstrated here underlines the need for a coherent approach to tracking live fish movements in GB.

This analysis is, to our knowledge, the first to assess the extent and relative importance of cross-border live fish movements within GB, although this has previously been highlighted in the context of BKD outbreaks that have affected farms on both sides of the border (Murray et al., 2012). Previous studies have also focused on the network of live fish movements, and not included the multiple network layers considered here. Our findings are consistent with previous studies on infection routes for spread of fish diseases (Murray et al., 2002; Taylor et al., 2010), in that the movement of live fish has the largest potential for spreading pathogens over long distances. This makes it the prime target for controlling disease spread, and fortunately, it is also the most amenable to controls. Movement restrictions have long been applied to prevent the spread of aquatic animal pathogens, dating back to the Diseases of Fish Act of 1937. An understanding of network structure has the potential to inform targeted surveillance to increase the efficiency of prevention and control strategies (Green et al., 2012; Enright and Kao, 2018) as part of a policy of risk-based surveillance (Stärk et al., 2006). In particular, our results show that large outbreaks are only possible when there is movement of live fish because the transport network is needed to make the network well-connected (Table 2). Without this, the extreme upper bound on outbreak size is 79 sites, although real outbreak sizes would be much smaller. A direct consequence is that an island-wide stoppage of live fish movements would isolate any outbreak to a handful of sites; however this would not be feasible in most cases as it would have more of an impact on the industry than the disease itself. Targeted movement controls, as per current policy (Anonymous, 2009a, b), are also potentially very effective but rely on rapid identification of infected sites and contact tracing.

We considered how easy it is to fragment the network by applying movement controls to nodes targeted according to network properties. We found that it is possible to identify a relatively small number of nodes with high influence due to their position in connecting sub-components of the network. More generally in infectious disease transmission, “super-spreaders” are often identified (Woolhouse et al., 1997), occurring due to high heterogeneity within a population, for example in the number of close contacts or in physiological or immunological factors associated with transmission efficiency (Stein, 2011). Our results suggest that, due to network contact heterogeneity alone, in the event of the introduction of a pathogen which could potentially infect all nodes on the network, 25 super-spreader nodes (0.7% of all nodes) could be responsible for 80% of the expected outbreak size, allowing for the possibility of effective targeted surveillance.

Our network analysis has revealed that there is relatively low risk of widespread epidemics on the transport network in isolation: as a measure of the expected size of an outbreak based on network properties alone, introduction of disease into a randomly-selected node on the network puts, on average, a relatively small proportion (3%) - of nodes at risk via transport links. However, the addition of other network layers increases this to over a fifth of the network nodes (21%). Interestingly, while fish farms dominate the network of potential disease transmission via the transport links, in the full multi-layer network, non-farm sites play a major role.

As discussed in the methods section, a number of assumptions were made when reconciling the two databases in order to account for the type of data recorded. We ignored potential transmission links upstream in rivers by upstream migration of wild fish and farm escapees. In Scotland, only movements recorded at both source and destination sites were included. These assumptions could lead to a conservative estimate of potential pathogen spread. Other assumptions may have overestimated the potential for transmission in a real outbreak: for example, our simple treatment of the potential for transmission locally up to a cut-off of 3 km ignores the anticipated decrease of transmission probability with distance as employed in diffusion kernel models (Jonkers et al., 2010). We considered the network as static, ignoring both the temporal pattern of fish movements and the dynamics of disease transmission, which is pathogen-specific. The analysis of network components gives conclusions on the potential for large outbreaks and how the network layers can combine to enable this. In this sense it represents a worst case scenario or statement of possibility. A more realistic assessment of the likely size of an outbreak and the potential effectiveness of control measures requires dynamic simulation of infectious disease transmission on this network.

In conclusion, the contact structure of GB’s salmon and trout aquaculture industry has been shown to be a single epidemiological unit due to cross-border live fish movement. The movement of live fish is the single most important network layer when considering the risk of large outbreaks, and should therefore remain the priority for control, with the potential for targeted surveillance of sites corresponding to high-centrality network nodes. Given the potential for even more widespread transmission via local, river and marine contacts, real outbreaks may be considerably greater than those anticipated by considering the transport network in isolation.

## Appendix A (Modelling marine links)

Coupled particle-hydrodynamic models are used to assess risks of transmission of pathogens between sites in aquaculture. Locally they have been used to assess transmission of the sea louse (L*epeophtheirus salmonis*) between salmon farms in Loch Linnhe (Salama et al., 2013) and Loch Fyne (Adams et al., 2012) and from these derive contact networks. In the absence of such a contact structure for the whole Scottish salmon farming regions a method of developing such a contact network was derived by the method described below.

As part of the licensing process, the Scottish Environment Protection Agency requires a minimum of 15 days of observations from water current meters located at the sea surface, cage-bottom and bed of a proposed fish farm site in order to model the deposition footprint of a new or modified site. These data contain observations at varying time intervals (*t*) between 10 and 20 min with observations of current direction (*D*) and speed *S* (ms^−1^) for each recording event. Fish farms are primarily located at the sea surface, therefore only the sea surface observations are utilised in the simplified modelling procedure. The farms have been licensed over a 30 year period and some of the records are missing. Of the 213 sites stocking fish in 2011, 35 required simulated data to be substituted. The simulation data is drawn from the Scottish Shelf Model (Price et al., 2016a,b,c) however six of these sites are located in areas where the model resolution was too low such as small embayments and are omitted from the network construction.

The location and consented biomass (*B_F_*) for each farm *F* is extracted from the FHI databases which are made publically available online at Scotland’s aquaculture website (SAW: see www.aquaculture.scotland.gov.uk).

The pathogen characteristics used to construct the network reflect the notifiable disease agent infectious salmon anaemia virus. The initial size of a viral cohort is the product of shedding rate (*r*) and farm biomass (*B_F_*). The maximum transit time (*t_max,F_*) of a shed viral cohort is determined for each farm by calculating the time taken until it reaches below the minimum infective dose φ = 10^−1^ TCID_50_ ml^-1^ kg^-1^ (Gregory et al., 2009). This is calculated using *r=*7.2 × 10^-1^ ml^-1^ h^-1^ kg^-1^ (Gregory et al., 2009) and pathogen decay rate λ = 0.12 h^-1^ (Løvdal and Enger, 2002):

$$t_{max,F}=\frac{-\ln\left(\frac{\varphi }{rB_{F}}\right)}{\lambda }$$

The location (northing (*Y*) and easting (*X*)) of each farm in Scotland is detailed in SAW allowing for a grid release position for all sites in Scotland. For each of the 213 farms, simulations were undertaken. Each simulation involved 1000 iterations whereby 1000 starting time points were randomly selected from the observational dataset of *D* and *S* taken at each farm. As *t* progressed, the currents experienced were assumed to be the next sequential observation condition.

The initial location (*X_0_,Y_0_*) of the particles is that of the release farm location: (*X_0_,Y_0_*)*=*(*X,Y*).

The *X* and *Y* location at each timestep is determined from the location at the previous timestep using a simplified discrete-time hydrodynamic term amended from Salama and Murray (2011) accounting for observed (or simulated) current directions and speeds:

$$X_{t+1}=X_{t}+S_{t}\cos\left(D_{t}\right)$$

$$Y_{t+1}=Y_{t}+S_{t}\sin\left(D_{t}\right)$$

The position of the particle is recorded at each timestep, enabling a calculation of the proportion of the total simulation time spent residing within a 500 m vicinity of a fish farm. This distance allows for variability and follows the methods of Adams et al. (2012) with regards to particle tracking modelling for estimating farm interconnectivity. If this time is greater than zero, then a marine link is specified indicating a potential transmission route.

For simplification, the model structure does not account for land. While this potentially means some unrealistic behaviour, the distances simulated are not large and most flow datasets are aligned parallel to the coast. It is assumed that the current conditions observed or simulated for a farm origin, will be consistent within the vicinity. That is to say, a particle located at the farm will have the same trajectory as a particle located at distance from the farm.
