# Supplementary material for: The contact structure of Great Britain’s salmon and trout aquaculture industry
Source: Epidemics. 2019 Sep;28:100342. doi: 10.1016/j.epidem.2019.05.001 (PMC6731520; doi:10.1016/j.epidem.2019.05.001)
Supplement: Supplementary file 1 [file mmc1.docx]

**The Contact Structure of Great Britain’s Salmon and Trout Aquaculture Industry: Supplementary tables and figures**

A. E. Jones^1^, L.A. Munro^2^, D.M. Green^3^, K.L. Morgan^1^, A.G. Murray^2^, R. Norman^3^ ,D. Ryder^4^, N.K.G. Salama^2^, N.G.H. Taylor^4^, M.A. Thrush^4^, I.S. Wallace^2^, K.J. Sharkey^1^

Affiliations:

1. University of Liverpool, U.K.

2. Marine Scotland Science, Aberdeen, U.K.

3. University of Stirling, U.K.

4. Centre for Environment, Fisheries and Aquaculture Science (Cefas), Weymouth, U.K.

**Supplementary tables**

Tables for England & Wales have prefix “E”, those for Scotland have prefix “S”, and those for mainland Britain as a whole have prefix “B.” Tables with number 1 refer to single-group nodes; those with number 2 refer to (single- or multi-group) sites. Tables with number 3 are split into part a covering transport *links*, and part b covering the frequency of recorded *movements* through these links. Lastly, Table B4 provides descriptive statistics for link distances per transmission type.

England and Wales (Cefas-administered part of the network)

**Supplementary Table E1**. Distribution of all English and Welsh salmonid aquaculture nodes, by group.

| Cefas salmonid nodes | Farm | Other | All |
| --- | --- | --- | --- |
| Salmon | 28 | 96 | 124 |
| Trout | 191 | 2162 | 2353 |
| Total | 219 | 2258 | 2477 |

**Supplementary Table E2**. Distribution of all English and Welsh salmonid aquaculture sites, by group.

| Cefas salmonid sites | Farm | Other | All |
| --- | --- | --- | --- |
| Salmon only | 5 | 83 | 88 |
| Trout only | 168 | 2149 | 2317 |
| Salmon & Trout | 23 | 13 | 36 |
| Total | 196 | 2245 | 2441 |

**Supplementary Table E3a**. The number of transport links in 2011-13, by group and destination type, within England and Wales.

| Cefas transport links | To-farm | To-other | All |
| --- | --- | --- | --- |
| Salmon | 34 | 82 | 116 |
| Trout | 590 | 2587 | 3177 |
| Total | 624 | 2669 | 3293 |

**Supplementary Table E3b**. The number of live fish movements in 2011-13, by group and destination type, in England and Wales.

| Cefas movements | To-farm | To-other | All |
| --- | --- | --- | --- |
| Salmon | 180 | 176 | 356 |
| Trout | 3667 | 5875 | 9542 |
| Total | 3847 | 6051 | 9898 |

Scotland (MSS-administered part of the network)

**Supplementary Table S1a**. Distribution of Scottish salmonid farm and fishery aquaculture nodes, by group (2009-2011).

| MSS salmonid nodes | Farm | Other | All |
| --- | --- | --- | --- |
| Salmon | 542 | 8 | 550 |
| Trout | 97 | 474 | 571 |
| Total | 639 | 482 | 1121 |

**Supplementary Table S2**. Distribution of Scottish salmonid sites, by group (2009-2011).

| MSS salmonid sites | Farm | Other | All |
| --- | --- | --- | --- |
| Salmon only | 498 | 7 | 505 |
| Trout only | 53 | 473 | 526 |
| Salmon & Trout | 44 | 1 | 45 |
| Total | 595 | 481 | 1076 |

**Supplementary Table S2b**. Distribution of Scottish salmonid inland and marine sites, by group (2009-2011).

| MSS salmonid sites | Inland | Marine | All |
| --- | --- | --- | --- |
| Salmon | 159 | 346 | 505 |
| Trout | 516 | 10 | 526 |
| Salmon & Trout | 32 | 13 | 45 |
| Total | 707 | 369 | 1076 |

**Supplementary Table S3a**. The number of within-Scotland transport links in 2009-11, by group and destination site type.

| MSS transport links | To-farm | To-other | All |
| --- | --- | --- | --- |
| Salmon | 876 | 7 | 883 |
| Trout | 119 | 667 | 786 |
| Total | 995 | 674 | 1669 |

**Supplementary Table S3b**. The number of live fish movements in 2009-11, by group and destination site type, in Scotland.

| MSS movements | To-farm | To-other | All |
| --- | --- | --- | --- |
| Salmon | 4171 | 11 | 4182 |
| Trout | 1475 | 9814 | 11289 |
| Total | 5646 | 9825 | 15471 |

**Supplementary Table S3c**. The number of Scottish transport links in 2009-11, by site water type and destination site type.

| MSS transport links | To-farm | To-other | All |
| --- | --- | --- | --- |
| Inland (freshwater) | 322 | 665 | 987 |
| Inland to Marine | 451 | 6 | 457 |
| Marine to inland | 26 | 3 | 29 |
| Marine (seawater) | 196 | 0 | 196 |
| Total | 995 | 674 | 1669 |

British Salmonid Aquaculture Industry Network

**Supplementary Table B1**. Distribution of British salmonid farm and fishery aquaculture nodes, by group.

| British salmonid sites | Farm | Other | All |
| --- | --- | --- | --- |
| Salmon | 570 | 104 | 674 |
| Trout | 288 | 2636 | 2924 |
| Total | 858 | 2740 | 3598 |

**Supplementary Table B2**. Distribution of British salmonid farm and fishery sites, by group.

|  | Farm | Other | All |
| --- | --- | --- | --- |
| Salmon only | 503 | 90 | 593 |
| Trout only | 221 | 2622 | 2843 |
| Salmon & Trout | 67 | 14 | 81 |
| Total | 791 | 2726 | 3517 |

**Supplementary Table B3a**. The number of cross-border links for 2009-11, by group and destination type.

| **Transport links from**  **England to Scotland** | To-farm | To-other | All |
| --- | --- | --- | --- |
| Salmon | 34 | 0 | 34 |
| Trout | 82 | 34 | 116 |
| Total | 116 | 34 | 150 |
| **Transport links from**  **Scotland to England** | To-farm | To-other | All |
| Salmon | 8 | 2 | 10 |
| Trout | 33 | 94 | 127 |
| Total | 41 | 96 | 137 |
| **All cross-border transport links** | To-farm | To-other | All |
| Salmon | 42 | 2 | 44 |
| Trout | 115 | 128 | 243 |
| Total | 157 | 130 | 287 |

**Supplementary Table B3b**. The number of cross-border movements for 2009-11, by group.

| \| **Movements from**  **England to Scotland** \|  \| \| --- \| --- \| \| Salmon \| 85 \| \| Trout \| 728 \| \| *Total* \| 813 \| \| ***Movements from***  ***Scotland to England*** \|  \| \| Salmon \| 67 \| \| Trout \| 1,097 \| \| Total \| 1,164 \| \| **All cross-border movements** \|  \| \| Salmon \| 152 \| \| Trout \| 1,825 \| \| Total \| 1,977 \| |  |  |  |
| --- | --- | --- | --- | --- | --- | --- | --- | --- | --- | --- | --- | --- | --- | --- | --- | --- | --- | --- | --- | --- | --- | --- | --- | --- | --- | --- | --- |

**Supplementary Table B4**. Network link distances (m) in the British salmonid network (2009-13).

| Link Type Layer | Number | Median | Mean | Max |
| --- | --- | --- | --- | --- |
| Local | 6250 | 1726 | 1641 | 3,000 |
| River | 839 | 12635 | 19672 | 123880 |
| Movement | 5249 | 53646 | 90142 | 793410 |
| Marine | 386 | 2443 | 3893 | 35895 |
| Overall | 12724 | 2789 | 39408 | 793410 |

**Supplementary figures**

| **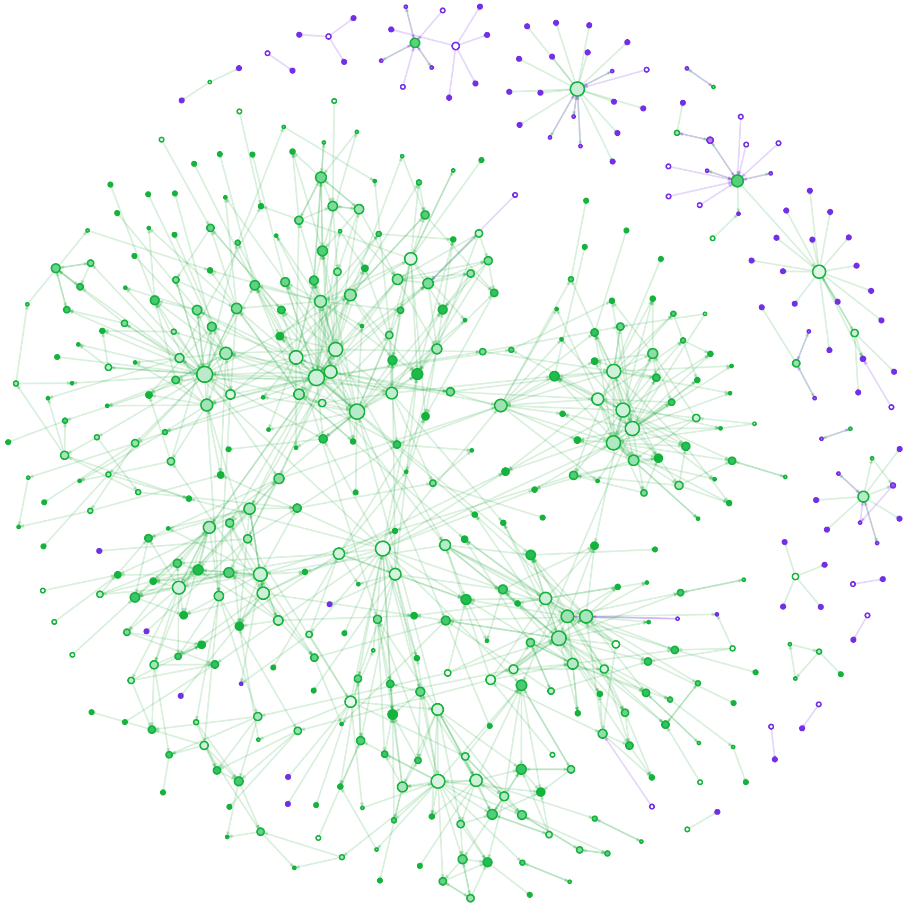** |
| --- |
| 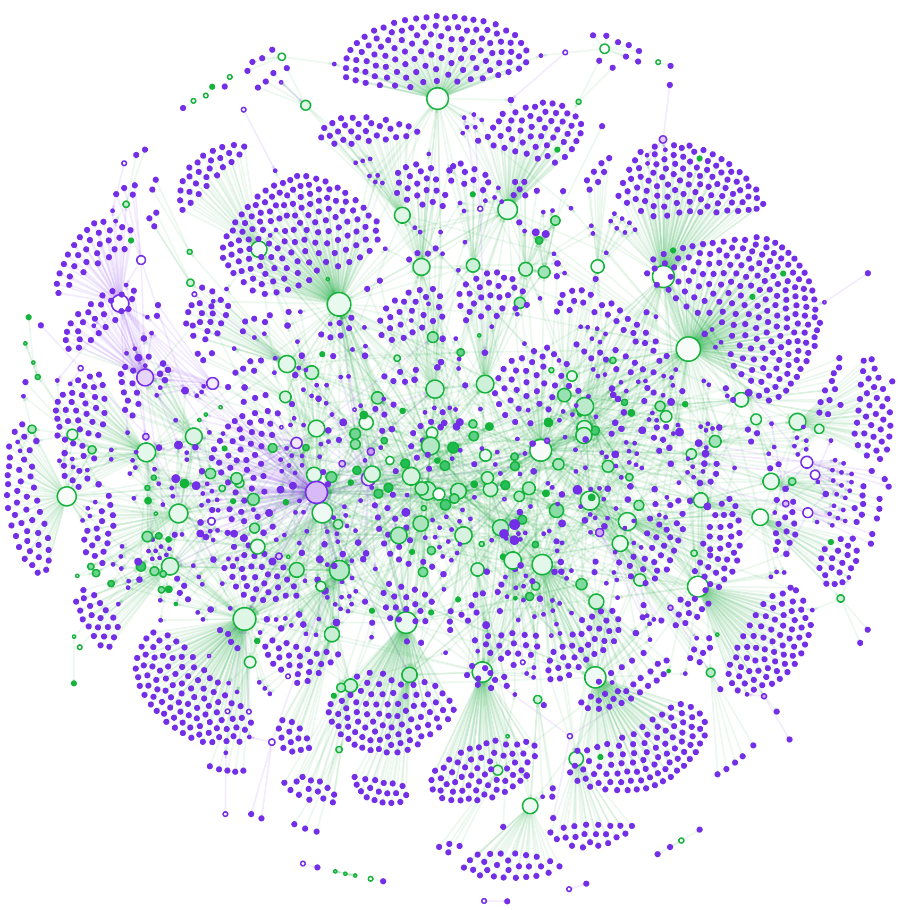 |
| **Figure S1** Plot of the British salmonid aquaculture industry transport links for salmon (top), and trout (bottom). Nodes designated as farms are colored green and other nodes blue. Node size is proportional to the log of the number of connections. Node color scales to white depending on proportion of connections for which node is the source (i.e. white = source, full color = sink). Links take the color of the source. |

| 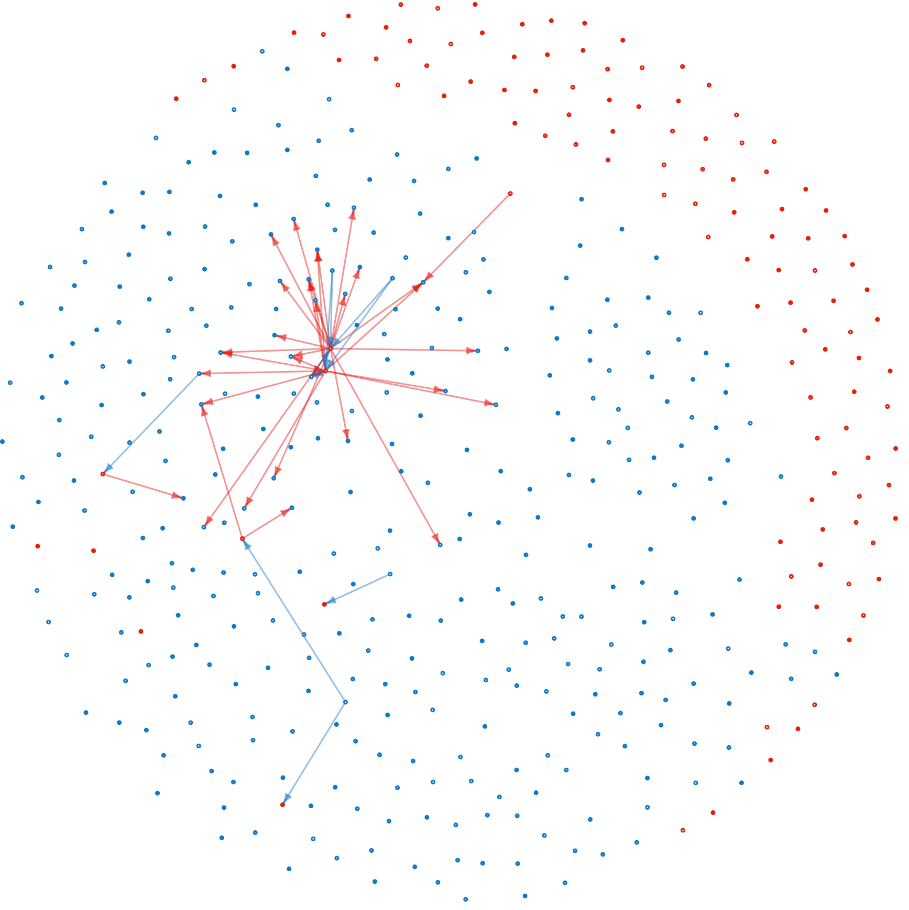 |
| --- |
| 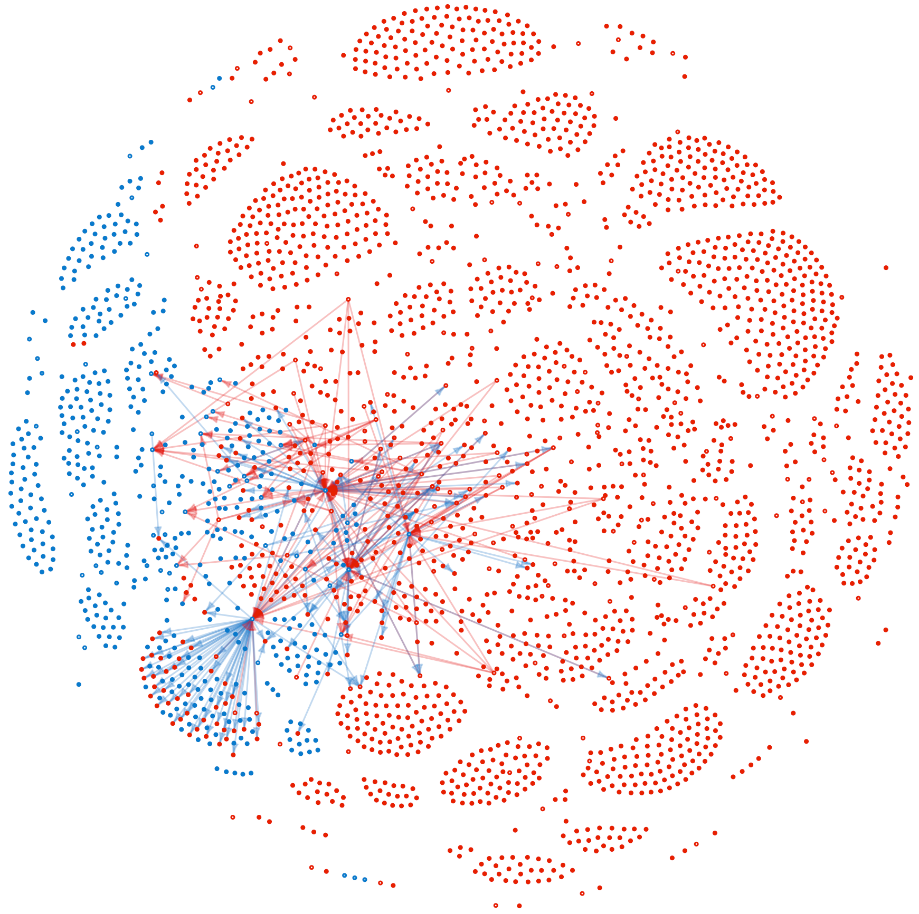 |
| **Figure S2** Plot of cross-border transport links for the British salmonid aquaculture industry networks for salmon (top), and trout (bottom). Nodes in the Marine Scotland network are colored blue and nodes in the Cefas network red. Links take the color of the source node. |

**
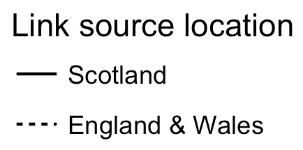

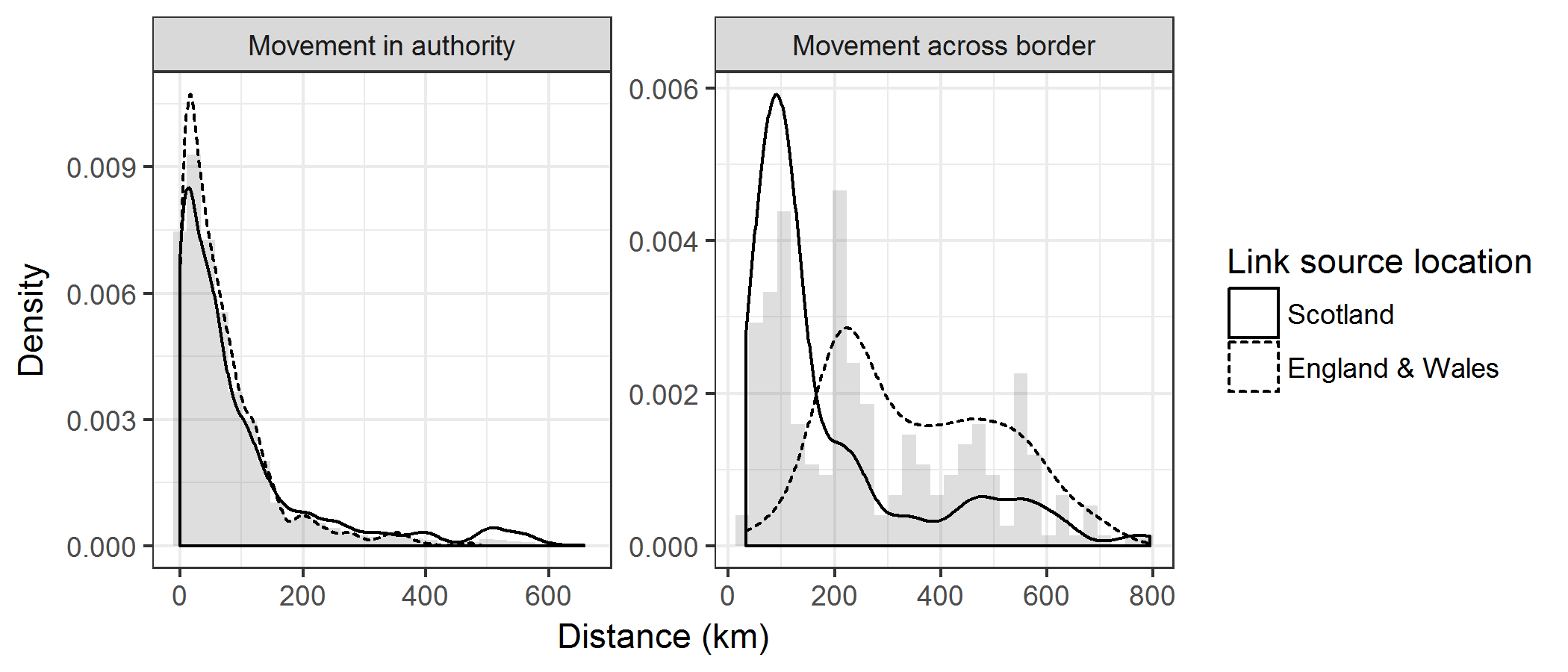
**

**Figure S3** Distribution of transport link distances in British salmonid network (km). Black lines given smoothed probability density for links originating in Scotland (solid) or England and Wales (dashed).
